# Supplementary material for: Single-Base Gene Variants in MIR-146A and SCN1A Genes Related to the Epileptogenic Process in Drug-Responsive and Drug-Resistant Temporal Lobe Epilepsy—A Preliminary Study in a Brazilian Cohort Sample
Source: Int J Mol Sci. 2024 May 30;25(11):6005. doi: 10.3390/ijms25116005 (PMC11172889; doi:10.3390/ijms25116005)
Supplement: Supplementary file 1 [file ijms-25-06005-s001.zip › ijms-2944597-supplementary.pdf]

Supplementary Table S1. microRNA (miR)-146a *(MIR-146A)* and Sodium Voltage-Gated Channel Alpha Subunit 1 (*SCN1A*) relative expression values for each evaluated sample.

rs2910164 – *MIR-146A*	Genotype	Samples	2-ΔΔCt	Genotype	Samples	2-ΔΔCt	Genotype	Samples	2-ΔΔCt
Drug-responsive	GG	SE03	2.80	GC	SE01	0.77	CC	SE09	2.11
	GG	SE05	2.77	GC	SE08	1.39	CC	SE12	0.85
	GG	SE13	3.56	GC	SE52	1.22	CC	SE35	1.75
	GG	SE16	1.96	GC	SE14	1.85	CC	SE42	2.60
	GG	SE17	0.88	GC	SE41	0.47			
	GG	SE19	0.02	GC	SE43	3.97			
Drug-resistant	GG	SE02	0.66	GC	SE04	0.04	CC	SE07	0.70
	GG	SE11	1.07	GC	SE10	0.12	CC	SE18	0.75
	GG	SE15	1.78	GC	SE32	0.03	CC	SE33	0.21
	GG	SE29	2.56	GC	SE37	0.33			
	GG	SE30	1.25						
	GG	SE66	0.25						
rs2298771 – *SCN1A*	Genotype	Samples	2-ΔΔCt	Genotype	Samples	2-ΔΔCt	Genotype	Samples	2-ΔΔCt
Drug-responsive	AA	SE09	1.34	AG	SE01	3.17	GG	SE08	2.21
	AA	SE12	1.13	AG	SE03	1.67	GG	SE25	2.16
	AA	SE13	0.45	AG	SE06	2.86	GG	SE70	3.36
	AA	SE14	0.71	AG	SE16	2.59			
	AA	SE17	1.57						
	AA	SE19	1.30						
Drug-resistant	AA	SE07	0.46	AG	SE02	0.78	GG	SE51	2.62
	AA	SE10	1.10	AG	SE04	2.45	GG	TE06	5.31
	AA	SE15	1.93	AG	SE11	1.35	GG	TE19	3.48
	AA	SE29	1.02	AG	SE30	1.54			
				AG	SE36	2.44			

ΔΔCt, delta-delta cycle threshold.
